# Anisotropic resonance modes emerging in an antiferromagnetic superconducting state

**DOI:** 10.1038/s41598-017-10208-1

**Published:** 2017-09-04

**Authors:** F. Waßer, C. H. Lee, K. Kihou, P. Steffens, K. Schmalzl, N. Qureshi, M. Braden

**Affiliations:** 1 II. Physikalisches Institut, Universität zu Köln, Zülpicher Str. 77, D-50937 Köln, Germany; 2 National Institute of Advanced Industrial Science and Technology (AIST), Tsukuba, Ibaraki 305-8568 Japan; 3 Institut Laue Langevin, 71 avenue des Martyrs, 38000 Grenoble, France; 4 Jülich Centre for Neutron Science, Forschungszentrum Jülich GmbH, Outstation at Institut Laue-Langevin, 71 avenue des Martyrs, 38000 Grenoble, France

## Abstract

Two strong arguments in favor of magnetically driven unconventional superconductivity arise from the coexistence and closeness of superconducting and magnetically ordered phases on the one hand, and from the emergence of magnetic spin-resonance modes at the superconducting transition on the other hand. Combining these two arguments one may ask about the nature of superconducting spin-resonance modes occurring in an antiferromagnetic state. This problem can be studied in underdoped BaFe_2_ As_2_, for which the local coexistence of large moment antiferromagnetism and superconductivity is well established by local probes. However, polarized neutron scattering experiments are required to identify the nature of the resonance modes. In the normal state of Co underdoped BaFe_2_ As_2_ the antiferromagnetic order results in broad magnetic gaps opening in all three spin directions that are reminiscent of the magnetic response in the parent compound. In the superconducting state two distinct anisotropic resonance excitations emerge, but in contrast to numerous studies on optimum and over-doped BaFe_2_ As_2_ there is no isotropic resonance excitation. The two anisotropic resonance modes appearing within the antiferromagnetic phase are attributed to a band selective superconducting state, in which longitudinal magnetic excitations are gapped by antiferromagnetic order with sizable moment.

## Introduction

The emergence of spin-resonance modes (SRM) in several superconductors is interpreted as key evidence for unconventional pairing mediated through magnetic fluctuations^1^. The resonance mode can be explained as a spin exciton, which requires a sign change of the superconducting (SC) gap. Neglecting spin-orbit coupling the SRM is expected to be isotropic in spin space, while for dominant spin-orbit coupling an Ising-type resonance mode arises, as it was indeed observed in the 4*f* compound CeCoIn_5_^2^.

In Fe-based superconductors^3–6^ the multi-band multi-orbital Fermi surface combined with medium-sized spin-orbit interaction results in a complex interplay. That spin-orbit coupling is non-negligible in Fe-based superconductors can be learned from the large anisotropy gaps in the magnon dispersion in the antiferromagnetic (AFM) parent materials^7^. In pure BaFe_2_ As_2_ the in-plane anisotropy is even larger than the out-of-plane one^7^, which points to a lifting of the tetragonal orbital degeneracy. Signatures of qualitatively the same spin-space anisotropy have been observed in the superconducting states in several FeAs-based superconductors. Polarized inelastic neutron scattering (INS) experiments on optimum Co-doped BaFe_2_ As_2_ show that two distinct resonance components appear at the SC transition, of which the lower one is anisotropic^8^ while a broader mode at larger energy remains isotropic. Similar experiments on Ni^9^ and K-optimum-doped BaFe_2_ As_2_^10^ have established qualitatively the same double resonance excitations as a general property of doped BaFe_2_ As_2_. Furthermore, even in K-overdoped BaFe_2_ As_2_^11^ an anisotropic additional low-energy SRM remains visible ruling out that it just arises from the presence of quasi-static magnetic order. In Co-doped NaFeAs^12, 13^ and in Na-doped BaFe_2_ As_2_^14^ the low-energy features even dominate the magnetic scattering in the SC state while there is only an anisotropic shoulder in K-overdoped BaFe_2_ As_2_^11^ and in LiFeAs^15^.

For optimum- and over-doped BaFe_2_ As_2_ the spin-space anisotropy of the lower SRM corresponds to the two magnetic soft axes of pure BaFe_2_ As_2_. These are along the in-plane component of the propagation vector^4–6^, along which moments align ([110] direction in the tetragonal lattice and *a* in the orthorhombic notation), and along *c*. In contrast the orthorhombic *b* direction forms the hard magnetic axis with the large anisotropy gap in the magnon dispersion^7^. This picture of two magnetically soft directions is nicely corroborated by the recent observation of the spin-reorientation transition in Na-doped BaFe_2_ As_2_, which leads to an AFM ordered moment along the *c* direction^14^.

Various models were proposed to explain the coexistence of anisotropic and isotropic resonance features. The low-energy resonance has been ascribed to quasi-static correlations while the isotropic resonance at higher energy is explained by the usual triplet exciton^16, 17^. In another approach the split resonance is attributed to an orbital and band selective pairing, which retains some well-defined orbital character in the low-energy resonance modes^18^.

**Figure 1:**
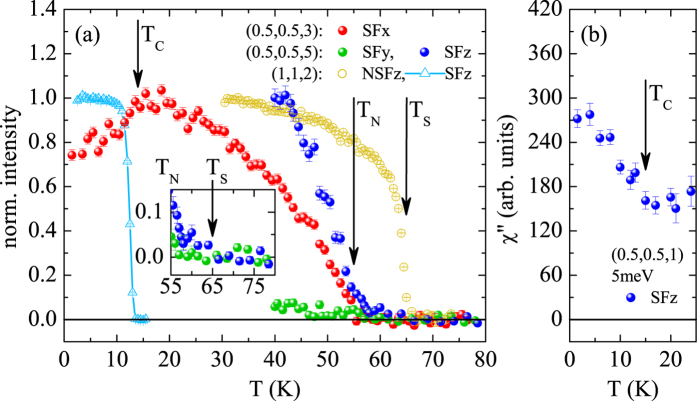
Temperature dependence of elastic and inelastic response in Ba(Fe_0.955_ Co_0.045_)_2_ As_2_. (**a**) The elastic response is used to define the three transition temperatures. In particular the nuclear Bragg peak at (1,1,2) exhibits a sharp intensity increase at the structural phase transition, *T*_*S*_ ∼ 65 K, while magnetic scattering at (0.5,0.5,3) and (0.5,0.5,5) appears at *T*_*N*_ ∼ 55 K. The SC transition of our sample crystal was studied through the neutron depolarization on the (1,1,2) nuclear Bragg peak yielding a *T*_*C*_ ∼ 14 K. The onset of superconductivity results in a suppression of the magnetic Bragg scattering at (0.5,0.5,3) as reported in^21^. Additionally, the persistence of anisotropic diffuse scattering in the spin-flip (SF) z- and y-channel in the nematic phase between *T*_*N*_ and *T*_*S*_ is displayed in the inset. Note, all the data has been normalized to their maximum values. (**b**) Below *T*_*C*_ the SRM is formed, as it can be seen in an increase of *χ*″ at (0.5,0.5,1) and 5 meV.

So far, polarized INS experiments on the spin-space anisotropy were performed only for near to optimum, or overdoped BaFe_2_ As_2_ with strongly suppressed AFM correlations (ordered moment of less than 5% of that in the pure material^19, 20^). Therefore, no sizable spin gap arising from the antiferromagnetic order can be expected, while the SC gaps are large. However, the phase diagram of Co-doped BaFe_2_ As_2_ offers the rather unique possibility to study the emergence of SRMs in a robust AFM state, which has not been fully explored in any of the unconventional superconductors^1^. In this work we study 4.5% Co underdoped BaFe_2_ As_2_ that exhibits microscopic coexistence of SC and AFM order, see Fig. 1, with an ordered magnetic moment of about 20% of that in the parent compound^19–22^. Many unpolarized INS studies on almost the same concentration were reported^21, 23–27^, revealing the appearance of a broad resonance feature near 4.5 meV, but only the polarized experiments presented here can show that the character of resonance modes in this AFM phase is fundamentally different from that at optimum or higher doping.

## Results

**Figure 2:**
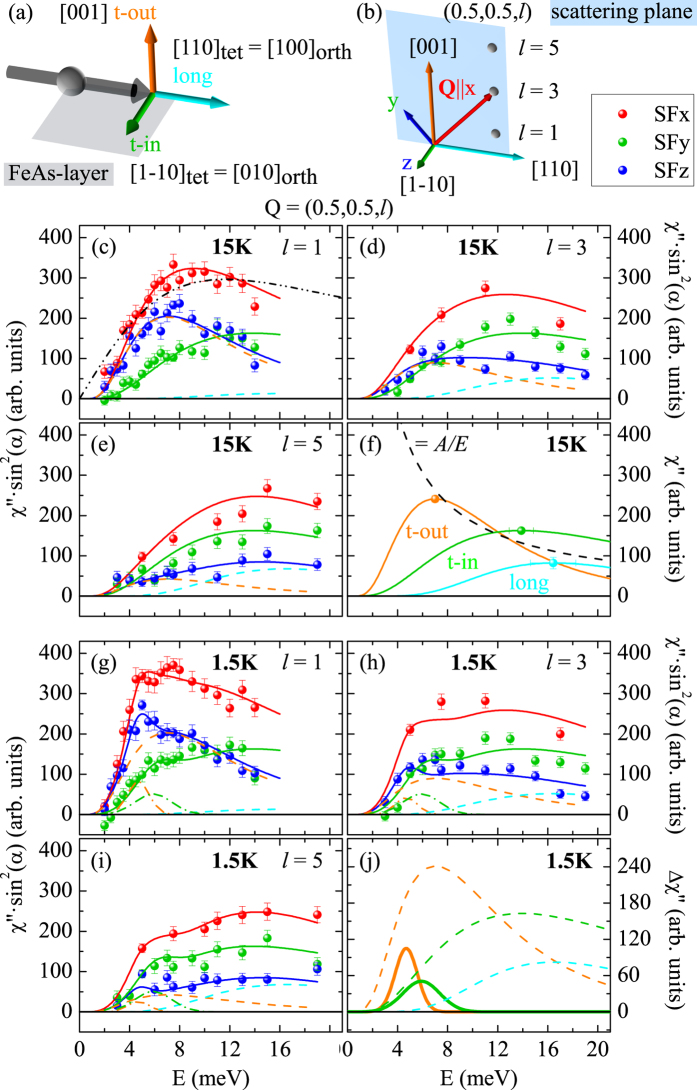
Magnetic fluctuation spectra in the AFM and SC states at various *l* values. (**a**) Sketch of the three orthogonal directions with respect to the ordered Fe moment. (**b**) Geometrical conditions for polarized INS at (0.5, 0.5, *l*) with respect to the spin-space anisotropy. (**c**–**e**) Polarized INS intensity at (0.5, 0.5, *l*) with *l* = 1, 3 and 5 in the different spin-flip (SF) channels after subtracting the background and correcting for the Bose and form factors. These data correspond to the dynamical susceptibilities *χ*″ multiplied with the geometry factors and folded with resolution function. The nine curves are consistently fitted (lines) by the three susceptibilities ${\chi^{\prime\prime}}_{long}$, ${\chi^{\prime\prime}}_{t-in}$ and ${\chi^{\prime\prime}}_{t-out}$ each described by a single log-normal distribution. These individual susceptibilities are resumed in (**f**); their amplitude follows an 1/*E* relation. A fit with a single relaxor function is not possible, as indicated by the dotted line in (**c**). (**g**–**i**) The same data in the SC phase at 1.5 K, where only two additional resonance components in ${\chi^{\prime\prime}}_{t-in}$ and ${\chi^{\prime\prime}}_{t-out}$ are needed to again consistently describe all nine curves. These additional resonance contributions are shown in (**j**), where dashed lines denote the anisotropic response in the normal state (same as in (**f**)).

Throughout this paper we use the tetragonal notation and performed all experiments in the [110]/[001] scattering plane. For the polarization analysis the frame of reference is defined as *x* parallel to the scattering vector *Q* (here all the magnetic scattering contributes to the spin flip (SF)x channel), *z* perpendicular to the scattering plane, and *y* perpendicular to *x* and *z*. This allows us to extract the contributions along the three directions in spin-space with respect to the ordered moment: longitudinal, $long\hat{=}{a}_{orth}$, transverse in-layer, $t-out\hat{=}{b}_{orth}$, and transverse out-of-layer, $t-out\hat{=}c$, see Fig. 2a.

Figure 1 resumes the various transitions appearing in Ba(Fe_0.955_ Co_0.045_)_2_ As_2_. The emergence of orthorhombic domains results in a lower crystal quality, and thus in a reduced extinction effect and enhanced Bragg intensity^8, 21^. Thereby we determine the structural transition to *T*_*S*_ ∼  65 K. The magnetic transition occurs at *T*_*N*_ ∼  55 K as seen in the sharp rise of the magnetic Bragg intensity. NMR, *μ* SR and previous neutron diffraction studies on samples of this concentration reveal an ordered moment of about 0.2 *μ*_*B*_ per Fe locally coexisting with the SC state^19–22^. Close inspection of the magnetic signals shows, that long-range intensity appears in the SFz channel, which agrees with the in-layer alignment of the moment. However, this anisotropy of SFz versus SFy persists in the diffuse scattering in the nematic phase between *T*_*N*_ and *T*_*S*_, see Fig. 1a. In this nematic phase the fourfold spin-space symmetry is already broken and the magnetic diffuse signal only corresponds to longitudinal in-layer correlations, *long* direction, that appear in SFz. The SC transition at 14 K, determined through neutron depolarization (see Methods and Fig. 1(a)), also leads to suppression of the magnetic intensity at the (0.5,0.5,3) magnetic Bragg peak by about 26% and to an increase of inelastic scattering at (0.5,0.5,1) and 5 meV, which corresponds to appearance of the resonance mode, see Fig. 1b, and the discussion below. All transition temperatures, the suppression of AFM moment at *T*_*c*_ and the emergence of SRMs perfectly agree with the literature^4–6, 21^.

By the aid of neutron-polarization analysis we are able to separate the magnetic fluctuations according to the three directions. Polarized INS scans at (0.5,0.5,*l*) with *l* = 1, 3 and 5 are shown in Fig. 2. These *Q* vectors correspond to AFM zone centers and the variation of the *l* values allows one to separate the *long* and *t* − *out* directions through the geometry factor sin^2^(*α*), see Methods. For small *l* the *c* or *t* − *out* direction is almost perpendicular to *Q*, so that the SFz signal essentially corresponds to the *t* − *out* direction. In contrast, at large *l Q* is nearly parallel to *c*, so that the SFz channel essentially contains the *long* direction. The *t* − *in* contribution exclusively contributes to the SFy channel with full geometry factor. The difference SFy + SFz-SFx gives a direct determination of the background, and 2SFx-SFy-SFz gives the total magnetic scattering besides the correction for finite flipping ratios^7^. Figure 2c–e shows the signal in the SF channels after subtracting the background. (The magnetic response also enters the nSFy and nSFz channels and can be obtained by nSFy-nSFx and nSFz-nSFx, and these data yield a fully consistent result, that, however, suffers from much lower statistics due to the larger nSF background). The data were corrected for higher-order contaminations of the monitor, the Bose factor and for the form-factor, so that they correspond to the imaginary part of the susceptibilities multiplied with the geometry factors, ${\sin }^{2}(\alpha )$, described above. The nine spectra can be consistently described by the three susceptibilities ${\chi^{\prime\prime}}_{long}$, ${\chi^{\prime\prime}}_{t-in}$ and ${\chi^{\prime\prime}}_{t-out}$. Each of these is well described by a log-normal distribution, ${A}_{i}{\exp }(-\frac{{(ln(E)-ln({\Gamma }_{i}))}^{2}}{2{\sigma }_{i}^{2}})$, which fits all spectra with only three parameters. In contrast to this satisfactory description, the magnetic spectra below *T*_*N*_ cannot be fitted by the relaxor function, $A\frac{E\cdot \Gamma }{{E}^{2}+{\Gamma }^{2}}$. The asymmetric spectral shape resulting from the instrumental resolution and from disorder-induced broadening is well captured by the log-normal distribution. By a concomitant fit of all spectra we obtain the three susceptibilities shown in Fig. 2f. The magnetic response of Ba(Fe_0.955_ Co_0.045_)_2_ As_2_ can be well understood as the spin-wave-like response of a magnetically ordered material with disorder^7, 21^. In the transversal channels *t* − *in* and *t* − *out*, the well-defined spin gaps of the pure material at 18.9 and 11.6 meV^7^ are renormalized to broad maxima at 13.9 and 7.0 meV. Also in the *long* channel we find a gap at ∼16 meV. In view of the report of a longitudinal gap in pure BaFe_2_ As_2_ of only 24 meV^28^, this would indicate a surprisingly small renormalization by 4.5% Co doping. In addition the strength of the longitudinal signal is comparable to that of the two transversal directions in the underdoped material, while much smaller longitudinal weight is reported in the pure crystal^28^. This sheds further doubts on the interpretation of the very weak signal in pure BaFe_2_ As_2_ as the longitudinal mode and supports an alternative two-magnon explanation^29^. By choosing the scattering vector (0.5, 0.5, *l*) in our sample crystal we select a single domain orientation for which the scattering vector constitutes a magnetic zone center, while it is a zone boundary for the other domain. Due to the very high magnon energies of the order of 200 meV, expected at the zone boundary^30^, we may exclude any scattering from the other domain, as it has recently been proven in an experiment on a detwinned crystal^31^.

**Figure 3:**
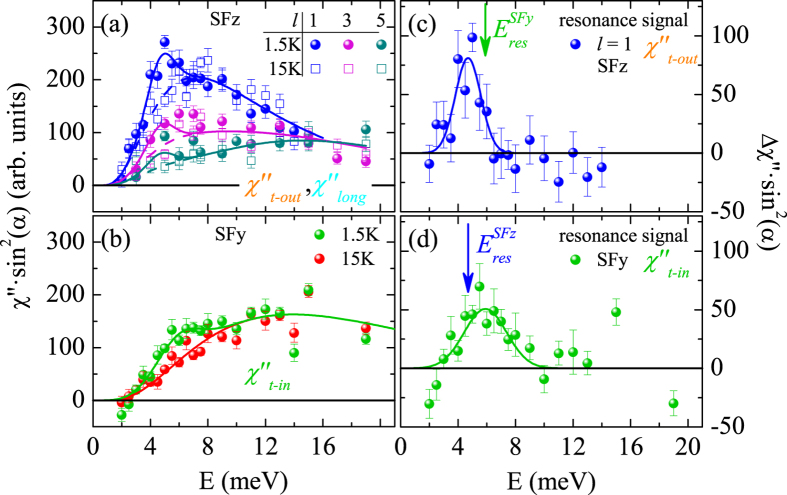
Emergence of two anisotropic spin resonance modes below *T*_*C*_. (**a**) SFz scattering at different *l* values with the analysis of Fig. 1b Comparison of SFy intensities obtained above and below *T*_*C*_; since this channel only contains ${\chi^{\prime\prime}}_{t-in}$, the results obtained at *l* = 1, 3 and 5 were added and lines correspond to the analysis presented in Fig. 1. The intensity enhancements in the SFz (**c**) and SFy (**d**) channels clearly peak at different energies indicating the different SRMs at 4.7 meV (${\chi^{\prime\prime}}_{t-out}$) and 5.9 meV (${\chi^{\prime\prime}}_{t-in}$).

**Figure 4:**
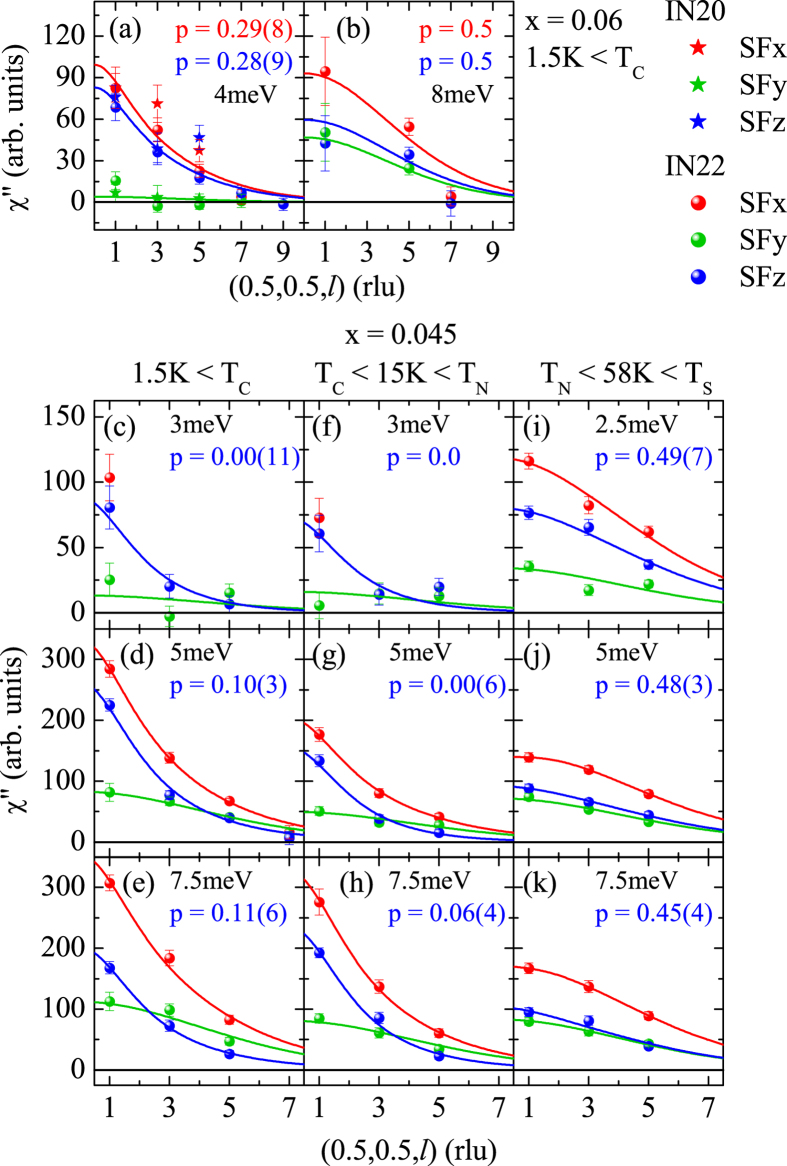
Polarization of resonance signals in 6% and in 4.5% Co-doped BaFe_2_ As_2_. *l* dependence of the scattering in various SF channels which allows one to separate the ${\chi^{\prime\prime}}_{long}$ and ${\chi^{\prime\prime}}_{t-out}$ components in the SFz channel. The parameter *p* describes the contribution of ${\chi^{\prime\prime}}_{t-out}$ required to fit the *l* dependence. Panel (a) shows the low temperature results for the 4 meV signal in optimum doped Ba(Fe_0.94_ Co_0.06_)_2_ As_2_, which has no *t* − *in* component but consists of 29% of *long* signal. In contrast, the isotropic resonance at ∼ 8 meV appears equally strong, *p* = 0.5, in the three directions (**b**). Panels (c–k) present the same analysis in Ba(Fe_0.955_ Co_0.045_)_2_ As_2_ for energies of 3, 5 and 7.5 meV, when not labelled differently, at T = 1.5 K in the SC state, at 15 K in the non-SC AFM phase, and at 58 K in the paramagnetic phase.

In Fig. 2g–i we present the same analysis of the magnetic scattering in the SC state. We can describe the nine spectra at the three studied scattering vectors by adding two resonance features, one in ${\chi^{\prime\prime}}_{t-out}$ and one in ${\chi^{\prime\prime}}_{t-in}$, to the fixed normal state susceptibilities determined at 15 K. That there are indeed two distinct resonance modes can be further seen in the SFy and SFz channels shown in Fig. 3a,b, respectively. The SFy only senses the ${\chi ^{\prime\prime}}_{t-in}$ with full geometry factor; therefore, we summed the data taken at the different *l* values. This ${\chi^{\prime\prime}}_{t-in}$ resonance peaks at 5.9 meV. In contrast, ${\chi^{\prime\prime}}_{t-out}$ and ${\chi^{\prime\prime}}_{long}$ contribute to the SFz channel with varying geometry factors (that always sum to one), see Fig. 2b. However, the ${\chi^{\prime\prime}}_{long}$ remains fully suppressed at energies below ∼8 meV, therefore the additional resonance signal in the SFz channels always stems from ${\chi^{\prime\prime}}_{t-out}$ and it peaks at 4.7 meV. The two resonance energies were obtained by a simultaneous fit of the spectra for *l* = 1, 3, and 5, at 1.5 K, see Fig. 2g–i by keeping the log-normal parameters identical to those determined at 15 K. Summing up all magnetic scattering at the three *l* values fully agrees with the unpolarized data taken previously^21, 23–26^. Our analysis can be corroborated by fitting the *l* dependence of the magnetic signals shown in Fig. 4. A larger *l* favors the observation of ${\chi^{\prime\prime}}_{long}$ on the dispense of ${\chi^{\prime\prime}}_{t-out}$ in the SFz channel. Therefore, we may determine the ratio *p* of the *long* component defined as $p=\frac{{\chi ^{\prime\prime} }_{long}}{{\chi ^{\prime\prime} }_{long}+{\chi ^{\prime\prime} }_{t-out}}$. At *T* = 58 K above the Néel temperature all signals yield *p* = 0.5 within the error bars, therefore there is no anisotropy between the two magnetically soft axes *long* and *t* − *out*, but the spectral weight along *t* − *in* (contributing to SFy) remains suppressed. The critical scattering of the AFM transition thus appears predominantly in the two soft directions, and small low-energy anisotropy remains even visible above *T*_*S*_ at 70 K. However, at 15 K in the AFM phase and at 1.5 K in the SC and AFM phase we find an insignificant *long* component in the SFz channel, *p* ∼ 0, in agreement with the conclusion that longitudinal excitations are gapped due to the significant ordered moment, see Fig. 4c–h. The same *l* analysis for the additional low-energy resonance signal in optimum-Co-doped BaFe_2_ As_2_ (x = 0.06) is shown in Fig. 4a. The slow reduction of the SFz signal with *l* indicates that there is a significant, *p* = 0.28, longitudinal component in this low-energy resonance mode at optimum doping; similar results were reported for optimum Ni doped^9^ and K overdoped^11^ BaFe_2_ As_2_.

## Discussion

The anisotropy of the resonance excitations in underdoped Ba(Fe_0.955_ Co_0.045_)_2_ As_2_ is fundamentally different from that seen in the near-optimum or overdoped BaFe_2_ As_2_ crystals studied previously by polarized INS experiments. This excludes a recently proposed generic explanation of the double resonance^27^. There is no isotropic SRM in the underdoped material, which can be associated with the usual spin triplet exciton^1^. Instead there are two polarized resonance modes appearing in the ${\chi^{\prime\prime}}_{t-out}$ and ${\chi^{\prime\prime}}_{t-in}$ channels. The reason for these fully anisotropic resonances seems to consist in the gap that opens in ${\chi^{\prime\prime}}_{long}$ due to the AFM ordering. Although this longitudinal gap is renormalized from the expected one in pure BaFe_2_ As_2_ (considering the optical studies^32, 33^) it is still considerably larger than twice the SC gap 2 Δ ∼ 8 meV determined in ARPES experiments^27^. Therefore, longitudinal fluctuations cannot interplay with the superconductivity in this underdoped material, and they cannot contribute to a lowering of exchange energy^1^. This yields a simple explanation for the reduced SC transition temperature although the high-energy longitudinal fluctuations still contribute to the pairing. The fact that we do not see longitudinal excitations in the SC state in contrast to the optimum or overdoped samples, furthermore, confirms that superconductivity and AFM ordering coexist locally for 4.5% Co-doping as it was reported by NMR and *μ* SR studies^19, 20^.

The anisotropic magnetic excitations are qualitatively compared in Table 1. At large temperatures the magnetic response is well described by a nearly isotropic relaxor behavior, which turns more and more anisotropic upon approaching the AFM and SC transitions in the underdoped and optimally doped compounds, respectively. Such an anisotropic relaxor response upon approaching a magnetic instability can be easily explained in random phase approximation, and has also been reported in Sr_2_ RuO_4_, which is close to a incommensurate magnetic ordering but not reaching it^34^. In both Co-doped compounds the softer relaxor functions are observed along the two soft magnetic axes. Entering the SC state directly (optimum doping) or through the AFM phase (underdoping) results, however, in different behaviors. For optimum doping the two soft magnetic directions of the normal state constitute the low-energy anisotropic SRM, while a second SRM at larger energy remains isotropic^8–11^

**Table 1:** Comparison of magnetic fluctuations in under- and optimum doped crystals. Comparison of the anisotropic magnetic fluctuations in the SC state with SRMs and in the normal state of under- and optimally doped BaFe_2_ As_2_.

	Superconducting		Normal state *T*_*C*_ < *T*
	SRM	character	
4.5% Under doped	4.7 meV	t – out	*T* < *T*_*N*_ gapped T_N_ ≲ T anisotropic relaxor *T*_*N*_ ≪ *T* isotropic relaxor
	5.9 meV	t – in	
6% Optimum doped	4 meV	28% long + 72%t − out	T_C_ ≲ T anisotropic relaxor *T*_*C*_ ≪ *T* isotropic relaxor
	9 meV	isotropic	

. In contrast, in the underdoped state the spin gaps induced by the AFM order dominate and result in SRMs appearing only in the two transverse channels. There is no isotropic SRM and the longitudinal channel does not contribute at all to SRMs in the AFM superconductor. Fundamentally different SRMs thus emerge in the under- and optimally doped samples as a result from the different phases from which SC emerges. A full explanation of the anisotropic SRMs thus needs to take the multi-orbital and multi-band structure into account as well as the selective SC pairing arising therein.

The emergence of SC in the AFM state of Ba(Fe_0.955_ Co_0.045_)_2_ As_2_ bears some resemblance to SC appearing in Fe-based compounds, in which the hole Fermi surfaces at the zone center are suppressed; nevertheless SC can be explained by spin fluctuations in such case^35^.

The anisotropic spin-resonance modes emerging in the antiferromagnetic and superconducting phase of Co-underdoped BaFe_2_ As_2_ reveal the high complexity of it’s superconducting state. While in the case of a conventional multi-band superconductor, such as MgB_2_^36^, different gaps develop on distinct bands, the addition of spin-orbit coupling and the coexistence of magnetic order put much higher constraints on the theoretical description of the superconducting state and mechanism. This physics is, however, not limited to the Fe-based superconductors, but is also relevant for various 4*f* and 5*f* systems, where the understanding of the electronic band structure seems less advanced. The spin-dependent band-structure of the antiferromagnetically ordered phase must form the theoretical basis, out of which the superconducting phase forms, but so far this band structure seems insufficiently known. Including spin-orbit coupling to the theory is also necessary, as otherwise the antiferromagnetic ordering would not open a gap in the transversal excitations. The strong gap in the longitudinal magnetic excitations arising from the AFM order is remarkable and prevents any coupling with superconductivity. This effect might give a clue to the superconducting mechanism, because the *T*_*c*_ is strongly reduced compared to the maximum value of 24 K appearing at only slightly higher doping.

In summary polarized INS experiments on the magnetic response in underdoped AFM Ba(Fe_0.955_ Co_0.045_)_2_ As_2_ reveals a fundamentally different behavior compared to previously studied compounds with a larger amount of doping. The significant ordered moment results in sizable spin gaps opening in the magnetic excitations along all three directions. Longitudinal fluctuations remain unaffected by the SC transition, because the corresponding AFM spin gap clearly exceeds twice the SC one. In the SC state two resonance components can be separated that are both anisotropic in spin space, one appearing in ${\chi^{\prime\prime}}_{t-out}$ the other in ${\chi^{\prime\prime}}_{t-in}$. In contrast the anisotropic low-energy SRM at optimum doping appears in the *long* and *t* − *out* directions. The consistent description of the anisotropic SRMs appearing in under- and optimally doped BaFe_2_ As_2_ certainly requires to take the detailed band- and orbital selective aspects of the SC pairing into account.

## Methods

Three single crystals of Ba(Fe_0.955_ Co_0.045_)_2_ As_2_ were grown by the FeAs-flux method yielding a combined mass of 2.12 g co-aligned for the experiment. The 6% Co-doped sample used for comparison is the same as that studied in ref. 8.

Polarized INS experiments were performed on the IN20 and IN22 thermal triple axis spectrometers at the Institut Laue-Langevin in Grenoble. Both spectrometers were operated with Heusler monochromator and analyzer crystals, and a graphite filter was set between the sample and the analyzer in order to suppress higher order contaminations. Most data was taken with the final wave vector of the neutron fixed to 2.662 Å^−1^. Experiments were performed with either the CRYOPAD device to assure zero magnetic field at the sample position or with Helmholtz coils to guide the neutron polarization at the sample^37^. With the Helmholtz coils the guide field was not varied in the SC state in order not to deteriorate the neutron polarization (except for the *T*_*c*_ measurement shown in Fig. 1b). The flipping ratio measured on nuclear Bragg peaks in the SC phase amounted to 14 on IN20 and 16 on IN22. Polarized INS allows one to separate magnetic and nuclear contributions and to split magnetic scattering according to the polarization direction of the magnetic signal^37^. In general INS only senses magnetic signals that are polarized perpendicular to the scattering vector **Q** resulting in a geometry factor sin^2^(*α*) with *α* the angle between **Q** and the magnetic signal. With the longitudinal polarization analysis this active part of the magnetic correlations further splits. In the neutron spin-flip (SF) channel one finds the part that is also perpendicular to the neutron polarization direction, while the neutron non-spin-flip (NSF) channel contains the parallel polarization^11^.

Varying the neutron-polarization guide fields in the SC state induces neutron depolarization, because the magnetic flux inside the sample is pinned. This depolarization induces a strong SF intensity on a non-magnetic Bragg peak. By following this *erroneous* intensity upon heating, the SC *T*_*c*_ = 14 K of the sample crystal is determined through the sharp drop of the spin-flip intensity associated with the depinning of guide fields and restoration of neutron polarization at *T*_*c*_, see Fig. 1b.
